# An Unsupervised Monocular Visual Odometry Based on Multi-Scale Modeling

**DOI:** 10.3390/s22145193

**Published:** 2022-07-11

**Authors:** Henghui Zhi, Chenyang Yin, Huibin Li, Shanmin Pang

**Affiliations:** 1 School of Mathematics and Statistics, Xi’an Jiaotong University, Xi’an 710049, China; 3120107031@stu.xjtu.edu.cn (H.Z.); 2264746043@stu.xjtu.edu.cn (C.Y.); 2 School of Software Engineering, Xi’an Jiaotong University, Xi’an 710049, China; pangsm@xjtu.edu.cn

**Keywords:** visual odometry, V-SLAM, unsupervised learning

## Abstract

Unsupervised deep learning methods have shown great success in jointly estimating camera pose and depth from monocular videos. However, previous methods mostly ignore the importance of multi-scale information, which is crucial for pose estimation and depth estimation, especially when the motion pattern is changed. This article proposes an unsupervised framework for monocular visual odometry (VO) that can model multi-scale information. The proposed method utilizes densely linked atrous convolutions to increase the receptive field size without losing image information, and adopts a non-local self-attention mechanism to effectively model the long-range dependency. Both of them can model objects of different scales in the image, thereby improving the accuracy of VO, especially in rotating scenes. Extensive experiments on the KITTI dataset have shown that our approach is competitive with other state-of-the-art unsupervised learning-based monocular methods and is comparable to supervised or model-based methods. In particular, we have achieved state-of-the-art results on rotation estimation.

## 1. Introduction

Visual odometry (VO) is the key part of V-SLAM, which can recover the camera’s 6-DOF pose and single-frame depth map from the video sequence. It is widely used in robotics [1], autonomous driving [2], augmented/virtual reality [3], and so on. Since VO has a clear definition in geometry, methods that are based on geometry and probability have been greatly developed, which are collectively referred to as model-based algorithms. According to the different methods of data association between adjacent frames, model-based algorithms can be grouped into feature-based and direct methods. After decades of progress, a large number of excellent algorithms have been proposed in each, such as ORB-SLAM [4] for the former group and LSD-SLAM [5], DSO [6] for the latter group. Despite their prosperity, model-based methods also have non-negligible shortcomings. First of all, the geometric probability models are based on static scene assumptions, but dynamic objects, such as people and cars, exactly exist in real scenes. Second, they only use the surface information of the image, while the deep semantics, space and other information are not well exploited. Third, they need complex manual procedures, such as sensor calibration, to complete the entire process [6]. When faced with challenging scenes, such as fast camera movement, lighting changes, and insufficient environmental textures, the algorithms will drop sharply or even fail to run.

As is well known, deep learning has achieved excellent results in many computer vision tasks, such as object detection, image classification, and semantic segmentation. This also has led researchers to employ deep learning in visual odometry. Supervised deep learning [7] regards VO as a regression problem. Overall, it utilizes a convolutional neural network (CNN) to extract efficient feature representations from raw RGB images, and then exploits a recurrent neural network (RNN) to regress 6-DOF camera motion. Supervised learning-based VO requires a large dataset with ground truth to train the networks. However, ground truth data are difficult and expensive to collect in practice. Thus, much attention is transferred to unsupervised learning.

Compare to supervised methods, unsupervised learning can achieve comparable performance [8,9] without requiring ground truth datasets. Unsupervised learning methods simultaneously estimate the camera pose and depth map of current frame, as well as reconstructing the adjacent frame by differentiable image warping. This process is usually achieved by constructing a loss function that measures the photometric consistency between the reconstructed and the real images. Since Zhou et al. [8] first introduced the concept of unsupervised learning, various approaches [10,11] have flourished successively, empowering the unprecedented flexibility and practicability of VO.

Although unsupervised methods have shown great progress in the monocular VO task, there is still room for improvement. For instance, the importance of multi-scale information for the visual odometry task was not noted in previous works. The multi-scale information is very critical for VSLAM and VO task because objects of different scales contribute differently to the motion. In particular, for small-scale objects, such as points, their relationship in the co-visible image is determined by the fundamental matrix, and it is more accurate to recover motion from the fundamental matrix in scenes of translational motion. For large-scale objects, such as lines and surfaces, the co-visible image not only contains the basic epipolar mapping relationship, but also has a homography geometric relationship. In the rotational motion scene, the fundamental matrix is degraded, so accurate motion estimation can be obtained by using the homography brought by large-scale objects. In the model-based algorithms, these can be accurately expressed using geometric modeling. However, in the learning-based algorithms, the CNN or RNN structure is limited by the size of the convolution kernel, and can only focus on the local information of the image. In other words, modeling the multi-scale information is a promise direction to improve the learning methods.

**Figure 1:**
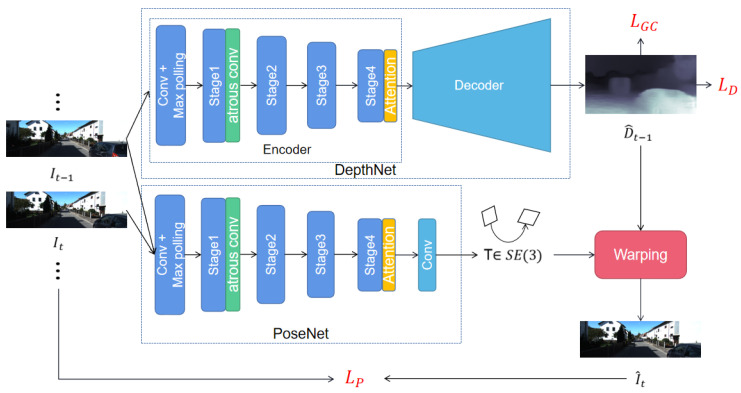
Illustration of our proposed framework. The DepthNet takes a single image as input and predicts corresponding depth map ${\hat{D}}_{t-1}$. The PoseNet takes every two consecutive images as input and predicts corresponding camera pose $T_{t-1,t}$. The differentiable image warping is applied to reconstructed image ${\hat{I}}_{t}$, then we calculate the photometric consistency loss $L_{P}$ according to $I_{t}$ and ${\hat{I}}_{t}$. The depth map ${\hat{D}}_{t-1}$ is used to calculate the depth loss $L_{D}$ and geometry consistency loss $L_{GC}$. Atrous conv denotes the densely linked atrous convolution layers, and attention denotes the non-local self-attention module.

Motivated by the aforementioned fact, we propose an unsupervised monocular VO framework based on multi-scale modeling. Our network consists of a pose estimation sub-network and a depth estimation sub-network, respectively, which is similar to the previous structure [7,8]. The pose estimation sub-network inputs adjacent image frames and regresses the relative transformation. The depth estimation sub-network inputs a single frame of image and outputs the corresponding depth map. The predicted pose and depth are then used to generate supervised information through the view synthesis technique, which guides the training of the entire network. The overall network framework is shown in Figure 1. As illustrated, our network develops two strategies to model the multi-scale information. First, we add dilated convolutional layers to the backbone of the network, which utilizes distant pixels to increase the size of the convolution kernel. In addition, we densely link convolutional layers with different dilation rates to increase the density of image pixels. As such, the size of the receptive field is significantly increased without losing image information. Second, we introduce a non-local self-attention mechanism, which computes the global dependencies between features at different locations, and then performs weighted fusion of the original feature maps. This mechanism makes each pixel in the feature map contain the information of other positions. In addition, we generate depth maps of different scales, and calculate the photometric loss separately at each scale to realize multi-scale information modeling at the image level. Our contributions can be summarized as follows:

1 We propose to use densely linked atrous convolutions to increase the receptive field size in VO task. As such, the network can effectively capture multi-scale information.
2 We propose to use the non-local self-attention mechanism to calculate the pixel-level pairwise relation as well as model the long-range dependency. Thus our network can make better use of the multi-scale information in the image.

We extensively evaluate the proposed framework on the KITTI dataset, and the results show that our method is competitive with other state-of-the-art unsupervised learning-based monocular methods and is comparable to supervised or model-based methods. In particular, we have achieved state-of-the-art results on rotation estimation.

The remainder of this article is organized as follows: Section 2 provides the research summary; Section 3 describes our architecture and the training scheme; Section 4 describes the experimental setting and illustrates the evaluation results with corresponding analysis; Section 5 offers concluding thoughts and directions for future work.

## 2. Related Work

### 2.1. Supervised Methods

PoseNet [12], proposed by Kendall et al. in 2015, is the first method that uses CNN to complete the VO task, which utilizes an end-to-end approach to directly regress the 6DOF camera poses from monocular image sequences. However, the VO gets the camera pose from consecutive image sequences, so researchers have turned their attention to the RNN network, which can better process sequence data. Wang et al. proposed DeepVO [7] in 2017, which uses a two-layer LSTM to process sequence information and realizes the learning of image sequence correlation. On the basis of this large framework, technologies such as optical flow estimation [13] and depth uncertainty [14] were introduced into VO, which further improves the accuracy and robustness. The limitation of supervised learning is that it requires a large amount of labeled data. The acquisition of ground truth often requires expensive equipment or highly manual labeling, and some gathered data are inaccurate. For example, depth obtained by LIDAR is sparse, and the output depth of Kinect contains a lot of noise. Furthermore, some ground truth is unable to be obtained (e.g., optical flow). Previous works have tried to address these problems with synthetic datasets [15], but there is always a gap between synthetic and real-world data.

### 2.2. Unsupervised Methods

Benefiting from datasets that do not require ground truth, unsupervised learning has better generalization and adaptability, so it has become the focus of research. In 2017, Zhou et al. [8] proposed to use a network capable of simultaneously predicting depth and pose, and used differentiable image warping to reconstruct the adjacent frame from the obtained depth and pose. The photometric loss was then calculated to guide the training of the network, and this framework has also become the basis for unsupervised methods. On this basis, Wang et al. [9] used optical flow as the network input, and used the consistency constraints of forward and backward optical flows to construct a loss function, which improved the accuracy of the network. Yasin, Li et al. [16,17] applied adversarial generative network (GAN) to further strengthen the ability to discriminate between the reconstructed image and the original image. In order to solve the problem of monocular scale uncertainty, UDeepVO [18] used the pose consistency constraints and binocular disparity smooth constraints to construct a loss function, which solves the scale uncertainty problem to a certain extent. GeoNet [19] proposed to use decomposed optical flow to eliminate dynamic objects in the scene, while the method proposed by Jia et al. [10] used the depth consistency constraint to form a mask to achieve the same purpose.

### 2.3. Atrous Convolution

Atrous convolution was first proposed in [20], which can efficiently compute wavelet transforms. It was first introduced into deep learning by Papandreou et al. [21], and it was also called dilated convolution [22]. Since then, dilated convolutions have been widely used in feature extraction modules in deep learning to obtain denser features. Atrous convolutions can also expand the receptive field of convolutional layers so that the obtained feature maps contain larger-scale information, which shows some advantages in semantic segmentation tasks [23]. Building on this approach, Yu et al. [22] used multiple atrous convolutional layers with different dilation rates to model the multi-scale context. In recent years, atrous convolution techniques have also been widely used in various deep deep learning tasks, such as object detection [24] and semantic segmentation [25]. In this paper, we introduce atrous convolution [26] into the VO task for the first time, and we use densely linked multi-layer atrous convolutions to capture multi-scale information in images.

### 2.4. Non-Loacl Self-Attention

Self-attention mechanisms have recently been successfully applied in various tasks, such as machine translation [27] and graph embedding [28]. Ref. [27] is one of the first attempts to apply a self-attention mechanism to model non-local dependencies in machine translation. NLNet [29] adopts self-attention mechanisms to model the pixel-level pairwise relations. CCNet [30] accelerates NLNet via stacking two criss-cross blocks, and is applied to semantic segmentation. However, NLNet actually learns query-independent attention maps for each query position, which is a waste of computation cost to model pixel-level pairwise relations. To model the global context features, SENet [31] rescales different channels to recalibrate the channel dependency with a global context. However, these methods adopt rescaling for feature fusion, which is not effective enough for non-local modeling. GCNet [32] via addition fusion as NLNet [29], with the lightweight property as SENet, is used to model the non-local global context. Inspired by these works, we propose to use a non-local self-attention block to effectively model multi-scale objects in images, thus improving the accuracy of VO.

## 3. Method

In this section, we introduce our method in detail. We first introduce the pipeline of our method illustrated in Figure 1. After that, we introduce the two proposed modules for modeling multi-scale information in Section 3.2 and Section 3.3, respectively. Finally, in Section 3.4, we introduce the loss function used for training.

### 3.1. Overview

Our method focuses on recovering the camera’s motion and depth of each frame from the monocular video. We consider the network that consists of a DepthNet and a PoseNet as the baseline. DepthNet is the structure of U-net [33] that utilizes ResNet50 [34] as the encoder. The decoder is composed by upsampling and deconvolution layers. PoseNet uses ResNet18 to extract the features, and regresses the 6-DOF relative transformation by 1×1 convolutional layers.

The input of the network is the continuous frames $\left\{I_{t-1},I_{t},\cdots ,I_{t+N}\right\}$. For simplicity, we only describe the processing process of two adjacent frames $\left\{I_{t-1},I_{t}\right\}$ of a video sequence, and multiple frames are similar. We take the concatenation of the current frame $I_{t}$ and the previous frame $I_{t-1}$ according to the channel as the input of PoseNet, and then regress the relative transformation $T_{t-1,t}\in \mathrm{SO}\left(3\right)$ between $I_{t-1}$ and $I_{t}$. DepthNet takes the previous frame $I_{t-1}$ as input and regresses the depth map ${\hat{D}}_{t-1}$. Then, we apply view synthesis to reconstruct $I_{t}$ by differentiable image warping:

(1) $$p_{t}∼KT_{t-1,t}{\hat{D}}_{t-1}\left(p_{t-1}\right)K^{-1}p_{t-1}$$

where $p_{t-1}$ and $p_{t}$ are the coordinates of a pixel in $I_{t-1}$ and $I_{t}$, respectively. *K* denotes the camera intrinsics. With the view synthesis described above, we obtain the reconstructed image ${\hat{I}}_{t}$. If the relative transformation $T_{t-1,t}$ and depth ${\hat{D}}_{t-1}$ are accurate enough, then the reconstructed image ${\hat{I}}_{t}$ and the real image $I_{t}$ should be the same, so we use the difference $∥{\hat{I}}_{t}-I_{t}∥$ as supervisory information to guide the optimization.

On this basis, we improve the DepthNet and PoseNet by integrating multi-scale information into the network. In particular, we use densely linked dilated convolution layers to increase the receptive field size for the encoders of DepthNet and PoseNet, and use a non-local self-attention mechanism to make the network notice pixel-level long-range dependencies.

### 3.2. Densely Connected Atrous Convolution

Atrous convolution that can increase receptive field while keeping the feature map resolution unchanged was first introduced in [23]. In the one-dimensional case, let y(i) denote the output signal and x(i) denote the input signal, and atrous convolution can be formulated as follows:

(2) $$y\left(i\right)=\sum_{k=1}^{T}x\left(i+d\times k\right)\times \omega \left(k\right)$$

where *d* is the dilation rate, ω(k) denotes the *k*-th parameter of filter, and *T* is the filter size. This equation reduces to a standard convolution when d=1. Atrous convolution is equivalent to convolving the input *x* with up-sampled filters produced by inserting d−1 zeros between two consecutive filter values. Thus, a large dilation rate means a large receptive field. For an atrous convolutional layer with the dilation rate *d* and the kernel size *T*, the equivalent receptive field size *R* is

(3) R=(d−1)×(T−1)+T

**Figure 2:**
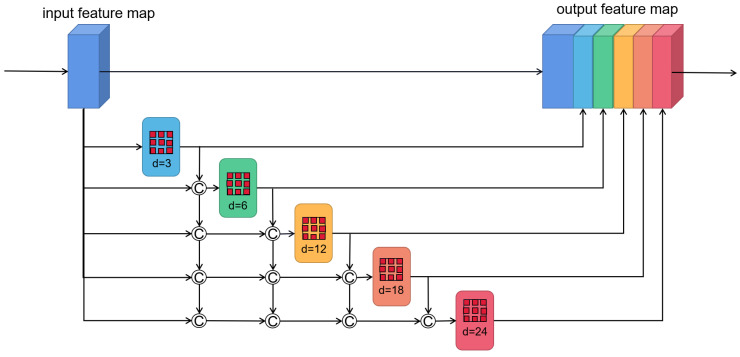
The structure of densely connected atrous convolution. The output of each dilated convolutional layer is concatenated with input feature maps, and then feed into the next dilated layer.

In the actual scene of VO, there are usually objects of different scales, which are very important for depth estimation and pose estimation. However, previous unsupervised methods simply used encoder to extract features for pose regression, resulting in the multi-scale information not being well modeled. To make use of multi-scale information, the feature maps must be able to cover different scales of receptive field. To this end, we add the densely linked atrous convolutional layers with different dilation rates [25] to the backbone of the encoder. The network details are shown in Figure 2. We cascade 5 convolutional layers with different dilation rates, and the smaller dilation rate is located in a lower layer. The input of this module is the feature maps, and the input for each atrous convolutional layer is the concatenation of the original feature maps and the previous atrous convolutional layers’ output. The formula is expressed below:

(4) $$y_{l}=H_{T,d_{l}}\left(\;\mathrm{concat}\;\left[y_{l-1},y_{l-2},...,y_{0}\right]\right)$$

where $H_{T,d_{l}}$ represents the atrous convolutional layer, $d_{l}$ represents the dilation rate of layer *l*, $y_{l}$ represents the output feature maps of layer *l*, and $\left[...\right]$ represents the concatenation operation. Finally, we concatenate the output of each atrous convolutional layer with the original feature maps as the final output of the entire module. Considering the influence of the gradual reduction of feature resolution, the entire module is embedded after stage1 of the encoder.

The advantages to densely link the atrous convolutional layers are double fold. First, it can not only make the receptive field significantly larger, but also can utilize more pixel information involved in feature extraction for large-scale objects. In the design, we set the kernel size be 3*3 for the atrous convolutional layer, and the dilation rates are 3, 6, 12 and 18, respectively. Therefore, the final receptive field size is

(5) $$\begin{array}{c} R_{\max}=R_{3,3}+R_{3,6}+R_{3,12}+R_{3,18}-3=79 \end{array}$$

**Figure 3:**
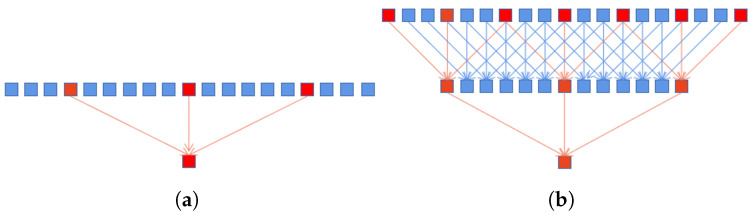
(**a**) Standard one-dimensional atrous convolution with dilation rate of 6. (**b**) Stacking an atrous convolution layer with different dilation rates.

This illustrates that the size of the receptive field almost reaches the size of the feature maps. Accordingly, the use of more pixels for modeling large-scale objects can be realized. Although the dilated convolution can effectively increase the size of the receptive field, the number of pixels used in the calculation process is the same as the standard convolution, which will lose a lot of pixel information. For example, the receptive field of size 13 contains only 3 pixels of information, as shown in Figure 3a. However, after densely linking, the input of a large dilation rate convolutional layer contains the output of the lower layers, as shown in Figure 3b. This is equivalent to first using a smaller dilation rate convolutional layer to compute the dense pixels, and then using the convolutional layer with a large dilation rate on this basis. Obviously, it leads to realizing the use of more pixels. The second benefit is that objects of different scales can be modeled. As shown in Figure 2, the final output of the module is obtained by concatenating the output of each dilated convolutional layer. Consequently, the output contains different level information, from small to large scales.

### 3.3. Non-Local Self-Attention

The non-local self-attention mechanism [29] is an effective way to make the current position contain the information of distant features by aggregating the information of other positions. Let $x={\left\{x_{i}\right\}}_{i=1}^{N_{p}}$ represent the input feature map of the non-local module, where $N_{p}$ is the number of positions in the feature map. Additionally, let $z={\left\{z_{i}\right\}}_{i=1}^{N_{p}}$ denote the output of a non-local module, which has the same dimensions as *x*. Thus, the calculation process of the entire module can be formulated as

(6) $$z_{i}=x_{i}+W_{z}\sum_{j=1}^{N_{p}}\frac{f\left(x_{i},x_{j}\right)}{C(x)}\left(W_{v}x_{j}\right)$$

where $f\left(x_{i},x_{j}\right)$ represents the relationship between the features $x_{i}$ and $x_{j}$, and C(x) is the corresponding normalization factor. $W_{z}$ and $W_{v}$ denote linear transform matrices (e.g., 1×1 convolution). For simplification, let $\omega_{ij}=\frac{f\left(x_{i},x_{j}\right)}{C(x)}$ represent the normalized relationship between $x_{i}$ and $x_{j}$, where the widely used form is embedded Gaussian, defined as $\omega_{ij}=\frac{\exp\left(<W_{q}x_{i},W_{k}x_{j}>\right)}{\sum_{m}\exp\left(<W_{q}x_{i},W_{k}x_{j}>\right)}$.

**Figure 4:**
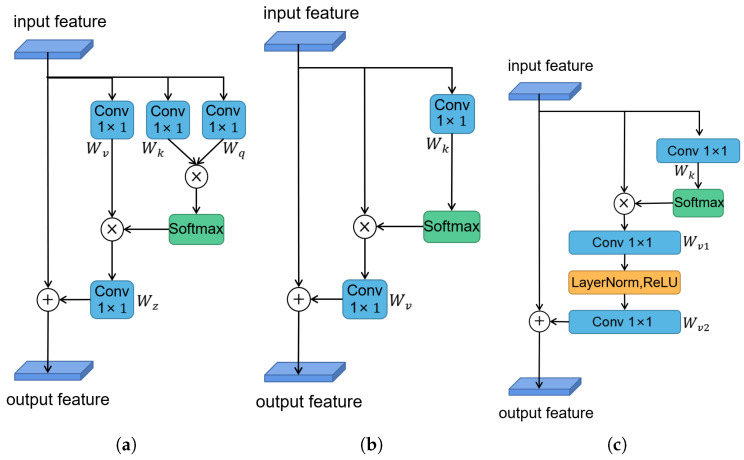
The structure of non-local self-attention. (**a**) The architecture of original non-local attention version (embedded Gaussian), (**b**) its simplified version, and (**c**) the final version that we used. ⊗ denotes matrix multiplication, ⊕ denotes broadcast elementwise addition.

The aforementioned non-local block can be regarded as a global context modeling block, which aggregates the information between the features of other positions and the current position. However, on the downside, this method has high time and space complexity, as it needs to calculate an attention map for each position. As a result, adding it directly to the network will dramatically slow down the training speed. In other words, it cannot be applicable for the real-time VO task. To reduce the problem, we calculate only a position-independent attention map based on the finding that attention maps corresponding to different positions are similar [32]. Thus, to improve the training and testing speed without losing too much accuracy, we simplify the formula as the following:

(7) $$z_{i}=x_{i}+\sum_{j=1}^{N_{p}}\frac{\exp\left(W_{k}x_{j}\right)}{\sum_{m}\exp\left(W_{k}x_{m}\right)}\left(W_{v}x_{j}\right)$$

where $W_{k}$ and $W_{v}$ denote linear transformation matrices. We show the simplified version of the non-local self-attention module in Figure 4b. Moreover, in order to further reduce the time complexity, we also apply the distributive law to move $W_{v}$ outside of the attention pooling

(8) $$z_{i}=x_{i}+W_{v}\sum_{j=1}^{N_{p}}\frac{\exp\left(W_{k}x_{j}\right)}{\sum_{m}\exp\left(W_{k}x_{m}\right)}\left(x_{j}\right)$$

At this time, the calculation complexity can be still large due to linear transform matrices $W_{k}$, which includes a 1×1 convolution with C×C parameters. In order to realize real-time processing, we replace the 1×1 convolution by a bottleneck transform module [31], which significantly reduces the number of parameters from C×C to 2×C×C/r, where *r* is the bottleneck ratio and C/r denotes the hidden representation dimension of the bottleneck. For instance, with default reduction ratio r=16, the number of parameters for transform module can be reduced to 1/8 of the original block. To ease optimization, we add layer normalization inside the bottleneck transform, which also can act as a regularizer that can benefit generalization. The final module structure is shown in Figure 4c, and the formula is as follows:

(9) $$z_{i}=x_{i}+W_{v2}\mathrm{ReLU}\left(LN\left(W_{v1}\sum_{j=1}^{N_{p}}\frac{\exp\left(W_{k}x_{j}\right)}{\sum_{m}\exp\left(W_{k}x_{m}\right)}x_{j}\right)\right)$$

where $W_{v1}$ and $W_{v2}$ denote linear transform matrices (e.g., 1×1 convolution), and LN denotes layer normalization.

As can be seen from the final formula, all positions on a channel of the feature map share the same weight, which is equivalent to weighting the channels. The module therefore has the ability to select features adaptively. It can select features that are appropriate for different movement patterns and thus make better use of the multi-scale information that has been learned to enhance the results. It is worth noting that the non-local module that can effectively model the global context information is very lightweight. Thus, to better capture the long-range dependency and select high-dimensional features, we embed the non-local module after stage4 of the encoder in DepthNet and PoseNet, which results in a slight increase in computation cost.

### 3.4. Loss Function

Appearance loss As explained in Section 3.1, if both the pose and depth estimation of our method are accurate enough, the reconstructed image ${\hat{I}}_{a}$ by differentiable warping should have the same appearance as the real image $I_{a}$. Therefore, we construct an appearance loss to measure the difference between them. The appearance loss is formulated as the following:

(10) $$L_{P}=\frac{1}{\left|V\right|}\sum_{p\in V}\left(\alpha ||I_{a}\left(p\right)-{\hat{I}}_{a}\left(p\right){\left|\right|}_{1}+\left(1-\alpha \right)\frac{1-{\mathrm{SSIM}}_{I_{a}{\hat{I}}_{a}}\left(p\right)}{2}\right)$$

where *V* stands for the set of points that are co-visible in images $I_{a}$ and ${\hat{I}}_{a}$, and *p* stands for a generic point in *V*. $\left|\right|\cdot {\left|\right|}_{1}$ stands for 1-norm, *a* is the timestamp, and α is the balance factor. ${\mathrm{SSIM}}_{I_{a}{\hat{I}}_{a}}\left(p\right)$ [35] is the structural similarity measure between images $I_{a}$ and ${\hat{I}}_{a}$, which measures the similarity between two images in terms of brightness, contrast and structure. This means that SSIM can better handle situations such as lighting changes. To be specific, the formula of SSIM is

(11) $$\mathrm{SSIM}\left(x,y\right)=\frac{\left(2\mu_{x}\mu_{y}+C_{1}\right)\left(2\sigma_{xy}+C_{2}\right)}{\left(\mu_{x}^{2}+\mu_{y}^{2}+C_{1}\right)\left(\sigma_{x}^{2}+\sigma_{y}^{2}+C_{2}\right)}$$

where x,y represents the 3*3 windows on the two images, respectively. $C_{1}$ and $C_{2}$ are constants. Additionally, μ and σ stand for the mean and variance of the image color, respectively.

Depth loss Because the depth and pose have a strong coupling relationship, the result of the depth estimation directly affects the authenticity of the reconstructed image. The discontinuity of depth usually happens where strong image gradients are present. To enforce discontinuity and local smoothness in depth, an edge-aware smoothness loss [10] is introduced. The formula is expressed as follows:

(12) $$L_{D}=\frac{1}{N}\sum_{x,y}∥\nabla_{x}\hat{D}\left(x,y\right)∥e^{-∥\nabla_{x}I\left(x,y\right)∥}+∥\nabla_{y}\hat{D}\left(x,y\right)∥e^{-∥\nabla_{y}I\left(x,y\right)∥}$$

where I(x,y) represents the image, and $\hat{D}\left(x,y\right)$ represents the predicted depth corresponding to I(x,y). *N* represents the size of the image.

Geometry consistency loss Because the depth predicted by the learning-based monocular VO method has per-frame scale ambiguity, there will be a scale-inconsistency issue in the results of long sequence videos, which affects the accuracy of the VO. For this reason, we introduce the geometric consistency loss proposed by [10]. For any two consecutive frames sampled from a video, we convert the predicted depth map in one frame to 3D space, then project it to the other frame using the estimated ego-motion. Finally, we minimize the inconsistency of the projected and the estimated depth maps. That is,

(13) $$L_{GC}=\frac{1}{\left|V\right|}\sum_{p\in V}\frac{\left|D_{b}^{a}\left(p\right)-D_{b}^{\prime }\left(p\right)\right|}{D_{b}^{a}\left(p\right)+D_{b}^{\prime }\left(p\right)}$$

where $D_{b}^{a}$ is the computed depth map of $I_{b}$ by warping $D_{a}$ using $T_{a,b}$, and $D_{b}^{{}^{\prime }}$ is the interpolated depth map from the estimated depth map $D_{b}$ (note that we cannot directly use $D_{b}$ because the warping flow does not lie on the pixel grid).

Finally, the overall loss function is

(14) $$Loss=\lambda_{1}L_{P}+\lambda_{2}L_{D}+\lambda_{3}L_{GC}$$

where $\lambda_{1}$, $\lambda_{2}$, $\lambda_{3}$ are trade-off parameters.

## 4. Experiments

In this section, we first introduce the implementation details of our method and the dataset, and then we perform a numerical comparison between our and other methods. Finally, we use ablation experiments to verify the effectiveness of each of our modules.

### 4.1. Implementation Details

The overall framework of our network is shown in Figure 1, which consists of DepthNet and PoseNet. For PoseNet, we use ResNet18 [34] to extract features, and we modify the first layer of ResNet18 to accept the concatenate image as input. Finally, we use four 1*1 convolutions layers to regress 6DOF relative transformation. DepthNet’s encoder is ResNet50, and the decoder adopts the structure of DispNet [36]. The input is a single-frame image, and the output is the depth map of four scales, where we calculate the loss on the four scales to improve the multi-scale learning ability of the network. For the activation function of the input layer, we use the sigmoid function, while for the activation function of all other layers, we use the ELU nonlinearities function.

Our method is implemented using the Pytorch framework on a single NVIDIA 3090 GPU. The two sub-networks are jointly trained through the loss. The network accepts three consecutive frames, and obtains the reconstructed images of the adjacent frames by warping the intermediate images. The input image is resized to 832 × 256 to balance accuracy and training time, and data augmentation, such as random scaling, cropping, and horizontal, is used to prevent over-fitting. We use the ADAM optimizer in training, and the decay rate is set to $10^{-4}$. The hyper-parameters $\lambda_{1}$, $\lambda_{2}$, and $\lambda_{3}$ in Equation (14) are 1, 0.1, and 0.5, respectively. We train 200 epochs with batch size = 4, and to ensure fast convergence, we use the pre-trained model on ImageNet [37].

The KITTI dataset [38] is used to train and evaluate the performance of the network. This dataset is currently the largest evaluation dataset for autonomous driving scenarios, which contains real image data collected from scenes such as urban areas, villages, and highways. There are, in total, 22 video sequences, of which 11 video sequences have ground-truth labels. The dataset is collected at a frequency of 10 Hz, where each sequence has up to 15 cars and 30 pedestrians with various degrees of occlusion and truncation.

### 4.2. Pose Estimation

We train the entire network using the 00–08 sequences and evaluate the pose estimation results using the 09–10 sequences. We measure our results using the standard measurement tools and translational and rotational errors are averaged over the entire sequence [39].

We compare our proposed method with some state-of-the-art learning-based methods, and the results are shown in Table 1. As monocular visual odometry has the scale ambiguity problem, we evaluate the monocular methods [8,9,10,11,19,40] after aligning with the ground truth. The basic framework for unsupervised monocular visual odometry was first proposed by SfMLearner [8]. On this basis, various methods further improve the accuracy and robustness of monocular VO by introducing optical flow auxiliary information [9], additional geometric constraints [10,11], RNN network structure [19], meta-learning [40], etc. However, they all ignore the important multi-scale information, and in contrast to them, our method achieves the state-of-the-art results because of its ability to model multi-scale information.To obtain scale-consistent results, methods such as [41,42,43] use baseline-corrected binocular image pairs for training. Compared to them, our method still achieves competitive performance. We also compare with the methods based on supervised learning [7,44,45,46]. Although these methods have simple network structure and fast training speed, they require ground truth to train the network. Compared to them, our method still has the lowest rotation error.

**Figure 5:**
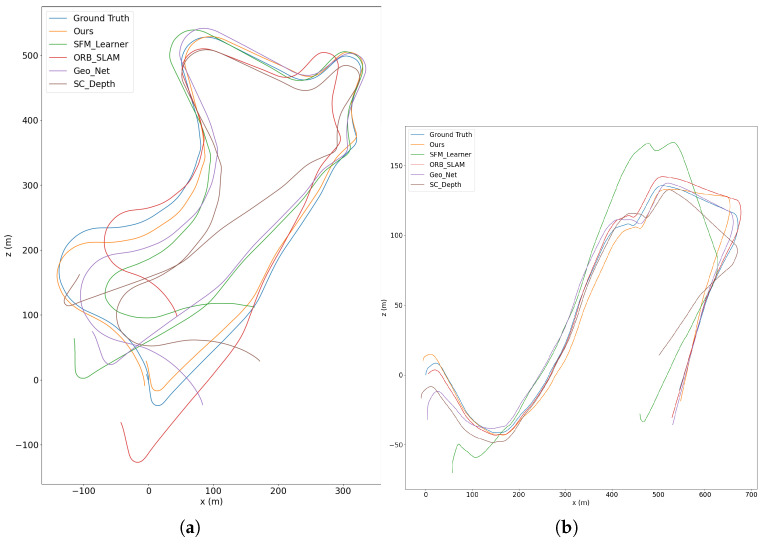
Trajectory results of different methods in sequence 09 (**a**) and sequence 10 (**b**) of KITTI dataset. Our method is much closer to the ground truth.

Finally, we compare with the classic traditional method ORB-SLAM [4], which has a strong back-end optimization system for improving the performance. As shown in the table, our method still has higher rotation accuracy than ORB-SLAM. Figure 5 shows a direct comparison of camera motion trajectories. As can be seen, our method is much closer to the ground truth. The comparison results confirm the key motivation of our method that leverages the multi-scale information contained in visual data. Thanks to the modeling of multi-scale objects, our method can exploit both the constraints on the fundamental matrix imposed by small-scale objects and the homography constraints imposed by large-scale objects. Thus we obtain excellent results not only in the translation scenes, but also in the rotation scenes where the fundamental matrix is degraded.

### 4.3. Depth Estimation

We take the split of Eigen et al. [47] to test our depth estimation. The ground truth used for testing is obtained by projecting the point cloud obtained onto a 2D plane with the light detection and ranging (LiDAR) sensor, where we interpolate the obtained depth map to the same size as the ground truth for comparison. As for evaluation metrics, we use the same evaluation tools as previous works [10], including the mean absolute relative error (AbsRel), mean log10 error (Log10), root mean squared error (RMS), root mean squared log error (RMSlog), and the accuracy under threshold ($\delta_{i}<1.25i,\;i=1,2,3$). These metrics provide a comprehensive evaluation of our depth estimation results. Because unsupervised monocular vision odometry cannot recover the absolute scale, when comparing to the ground-truth, we multiply the acquired depth map by the scale factor to obtain a same median of ground truth.

**Figure 6:**
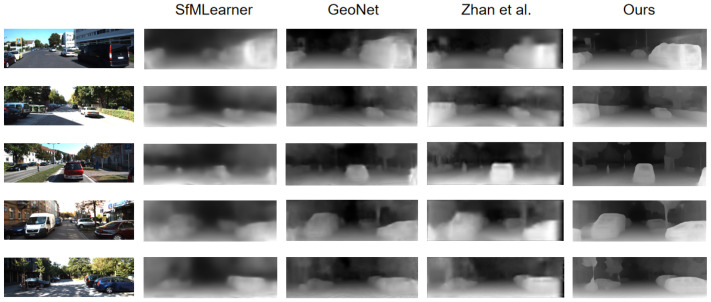
Visual comparison of the estimated depth maps on the KITTI Eigen test set. Our method shows better prediction on objects of different scales, low texture regions and clearly in both close and distant areas.

Table 2 shows the comparison results with other methods. Compared to unsupervised monocular depth estimation methods [8,10,19,48], our method achieves the highest accuracy, even if [19,48] jointly learn multiple tasks. Compared to the supervised methods [18,43,49] that use depth supervision or calibrated stereo images, our method is still quite competitive. To better understand comparison results, we visualize an example in Figure 6. As shown, our method can better predict the depth of cars and other multi-scale objects than other methods. This again demonstrates the importance to strengthen the multi-scale information.

### 4.4. Ablation Study

To demonstrate the effectiveness of each module, we conduct ablation experiments and compare the experimental results. Our baseline is similar to the method [10], which includes two sub-networks, PoseNet and DepthNet. The loss function is also the same as that used in Section 3.4. We add the proposed modules into the baseline and consequently evaluate the pose estimation results. The results of pose estimation are shown in Table 3.

**Figure 7:**
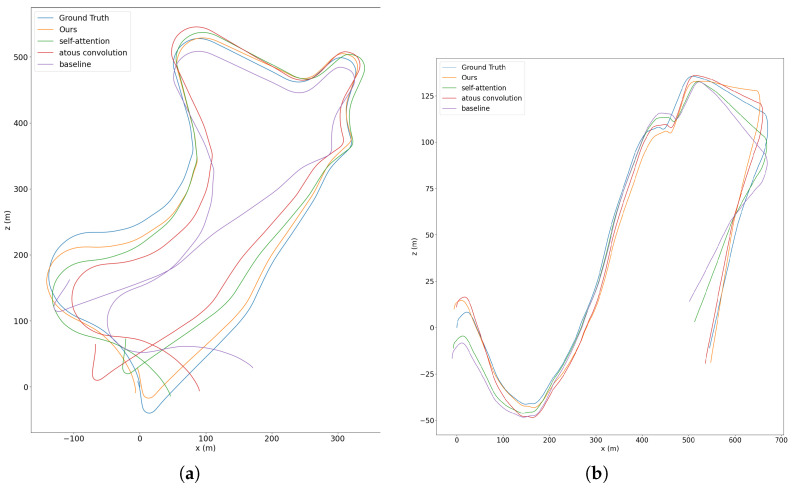
Trajectory results of ablation studies in sequence 09 (**a**) and sequence 10 (**b**) of KITTI dataset.

We first evaluate the baseline. Due to the joint training of the two sub-networks and the use of depth geometry consistency, our baseline method also outperforms other methods. We then add the atrous convolutions module. Since we densely linked atrous convolutional layers with a different dilation rate, the extracted features contain information at various scales that is useful for motion estimation. We can see from Table 3 and Figure 7 that there is some improvement in accuracy for both translations and rotations. We next add the non-local self-attention module. Because the non-local self-attention module can calculate the pixel-level pairwise relation as well as model the long-range dependency, thus our network can make better use of the multi-scale information in the image. In addition, our simplified self-attention mechanism can weight the channels of the feature map, meaning that it can automatically select features that are more suitable for translation or rotation, thus improving accuracy. Finally, we add both the atrous convolutions and the non-local self-attention module. As shown in Table 3 and Figure 7, the experimental results are further enhanced by the simultaneous use of the two modules. We guess that this is because the features extracted by the network contain more multi-scale information, which further contributes to the motion estimation.

## 5. Conclusions

In this paper, we propose a novel unsupervised deep learning method for pose estimation and depth estimation from monocular video. We use densely linked atrous convolutional layers to model multi-scale objects in images, and use a non-local attention mechanism to learn long-range dependencies in images. Both of these modules enable our network to better utilize multi-scale information, thereby improving the performance of depth estimation and pose estimation. Extensive experiments have proven that our method achieves competitive results in monocular visual odometry. In particular, we achieve state-of-the-art accuracy in rotation estimation. Our results are still quite competitive, even compared with supervised methods, stereo methods, and model-based methods. In the future, we plan to employ the domain generalization or domain adaptation techniques to improve the performance of our method on datasets that are different from the training scenarios. In addition, we plan to extend our method to the complete SLAM algorithm, including back-end optimization, dense mapping and other steps, and truly apply it to the fields of autonomous driving, AR, and robotics.

## Figures and Tables

**Table 1:** Visual odometry results on KITTI dataset. Mono/Ste stands for training on monocular/stereo videos, Su stands for supervised learning method, and Ge stands for model-based method. The underline represents the best result among all types of methods, and the boldface represents the best result among the monocular methods.

Method	Type	Seq.09		Seq.10	
		$t_{err}\;\left(\%\right)$	$r_{err}\;\left(\%/100\;m\right)$	$t_{err}\;\left(\%\right)$	$r_{err}\;\left(\%/100\;m\right)$
SfMLearner [8]	Mono	19.15	6.82	40.40	17.69
GeoNet [19]	Mono	28.72	9.8	23.90	9.0
Wang et al. [9]	Mono	9.88	3.40	12.24	5.2
DeepMatchVO [11]	Mono	9.91	3.8	12.18	3.9
SC-Depth [10]	Mono	7.31	3.05	7.79	4.9
Depth-VO-Feat [41]	Ste	11.89	3.6	12.82	3.41
Shunkai Li et al. [42]	Ste	6.23	2.11	12.9	3.17
Xiangyu Li et al. [43]	Ste	2.26	1.06	3.00	1.28
DeepVO [7]	Su	5.96	6.12	9.77	10.20
WPO-Net [46]	Su	8.19	3.02	8.95	3.12
Fei Xue et al. [44]	Su	3.47	1.75	3.94	1.72
DAVO [45]	Su	3.91	1.46	5.37	1.64
ORB-SLAM2-M (w/o LC) [4])	Ge	9.67	0.3	4.04	0.3
ORB-SLAM2-M [4]	Ge	3.22	0.4	4.25	0.3
Ours	Mono	7.58	**0.51**	**7.35**	**1.35**

**Table 2:** Single-view depth estimation results on KITTI odometry split. Mono stands for training on stereo videos, Ste stands for supervised learning using calibrated stereo images. The boldface represents the best result among all methods.

Method	Supervision	Error				Accuracy		
		AbsRel	SqRel	RMSE	RMSE log	δ<1.25	$\delta <1.25^{2}$	$\delta <1.25^{3}$
Zhou et al. [8]	Mono	0.208	1.768	6.856	0.283	0.678	0.885	0.957
GeoNet [19]	Mono	0.155	1.296	5.857	0.233	0.793	0.931	0.973
CC [48]	Mono	0.140	1.070	5.326	0.217	0.826	0.941	0.975
SC-Depth [10]	Mono	0.137	1.089	5.439	0.217	0.830	0.942	0975
Li et al. [18]	Ste	0.183	1.73	6.57	0.268	–	–	–
Godard et al. [49]	Ste	0.148	1.344	5.927	0.247	0.803	0.922	0.964
Zhan et al. [41]	Ste	0.144	1.391	5.869	0.241	0.803	0.928	0.969
Pilzer et al. [50]	Ste	0.1424	1.2306	5.785	0.239	0.795	0.924	0.968
Wong et al. [51]	Ste	0.135	1.157	5.556	0.234	0.820	0.932	0.968
Xiangyu Li et al. [43]	Ste	0.135	1.234	5.624	0.233	0.823	0.932	0.968
Ours	Mono	**0.125**	**0.992**	**5.192**	**0.208**	**0.844**	**0.947**	**0.977**

**Table 3:** Ablation study for various versions of our method on KITTI sequence 09 and 10. The least error results are highlighted in bold text. The boldface represents the best result.

Method	Seq.09		Seq.10	
	$t_{err}\;\left(\%\right)$	$r_{err}\;\left(\%/100\;m\right)$	$t_{err}\;\left(\%\right)$	$r_{err}\;\left(\%/100\;m\right)$
baseline	14.43	4.23	10.93	2.53
atrous	9.14	1.15	10.17	1.54
attention	7.67	0.69	8.16	1.55
baseline + atrous + self-attention	**7.58**	**0.51**	**7.35**	**1.35**

## Data Availability

The KITTI Dataset [38] used for this study can be accessed at http://www.cvlibs.net/datasets/kitti/ (accessed on 27 January 2021).

## References

[1] DeSouza G.N., Kak A.C. (2002). Vision for mobile robot navigation: A survey. IEEE Trans. Pattern Anal. Mach. Intell..

[2] Chen C., Seff A., Kornhauser A., Xiao J. Deepdriving: Learning affordance for direct perception in autonomous driving. Proceedings of the IEEE International Conference on Computer Vision.

[3] Azuma R.T. (1997). A survey of augmented reality. Presence Teleoperators Virtual Environ..

[4] Mur-Artal R., Montiel J.M., Tardos J.D. (2015). ORB-SLAM: A Versatile and Accurate Monocular SLAM System. IEEE Trans. Robot..

[5] Engel J., Schops T., Cremers D. LSD-SLAM: Large-scale direct monocular SLAM. Proceedings of the European Conference on Computer Vision.

[6] Engel J., Koltun V., Cremers D. (2017). Direct sparse odometry. IEEE Trans. Pattern Anal. Mach. Intell..

[7] Wang S., Clark R., Wen H., Trigoni N. Deepvo: Towards end-to-end visual odometry with deep recurrent convolutional neural networks. Proceedings of the 2017 IEEE International Conference on Robotics and Automation (ICRA).

[8] Zhou T., Brown M., Snavely N., Lowe D.G. Unsupervised learning of depth and ego-motion from video. Proceedings of the IEEE Conference on Computer Vision and Pattern Recognition.

[9] Wang R., Pizer S.M., Frahm J.M. Recurrent neural network for (un-) supervised learning of monocular video visual odometry and depth. Proceedings of the IEEE/CVF Conference on Computer Vision and Pattern Recognition.

[10] Bian J.W., Zhan H., Wang N., Li Z., Zhang L., Shen C., Cheng M.M., Reid I. (2021). Unsupervised Scale-consistent Depth Learning from Video. Int. J. Comput. Vis..

[11] Shen T., Luo Z., Zhou L., Deng H., Zhang R., Fang T., Quan L. Beyond photometric loss for self-supervised ego-motion estimation. Proceedings of the 2019 International Conference on Robotics and Automation.

[12] Kendall A., Grimes M., Cipolla R. Posenet: A convolutional network for real-time 6-dof camera relocalization. Proceedings of the IEEE International Conference on Computer Vision (ICCV).

[13] Pandey T., Pena D., Byrne J., Pandey T., Moloney D. (2021). Leveraging deep learning for visual odometry using optical flow. Sensors.

[14] Costante G., Mancini M. (2020). Uncertainty Estimation for Driven Visual Odometry. IEEE Trans. Robot..

[15] Dosovitskiy A., Fischer P., Ilg E., Hausser P., Hazirbas C., Golkov V., van der Smagt P., Cremers D., Brox T. Flownet: Learning optical flow with convolutional networks. Proceedings of the IEEE International Conference on Computer Vision (ICCV).

[16] Almalioglu Y., Saputra M.R.U., de Gusmao P.P.B., Markham A., Trigoni N. Ganvo: Unsupervised deep monocular visual odometry and depth estimation with generative adversarial networks. Proceedings of the 2019 International Conference on Robotics and Automation (ICRA).

[17] Li S., Xue F., Wang X., Yan Z., Zha H. Sequential adversarial learning for self-supervised deep visual odometry. Proceedings of the IEEE/CVF International Conference on Computer Vision.

[18] Li R.H., Wang S., Long Z.Q., Gu D.B. Undeepvo: Monocular visual odometry through unsupervised deep learning. Proceedings of the 2018 IEE International Conference on Robotics and Automation (ICRA).

[19] Yin Z.C., Shi J.P. Geonet: Unsupervised learning of dense depth, optical flow and camera pose. Proceedings of the IEEE Conference on Computer Vision and Pattern Recognition.

[20] Holschneider M., Kronland-Martinet R., Morlet J., Tchamitchian P., Tchamitchian P. (1989). A real-time algorithm for signal analysis with the help of the wavelet transform. Wavelets: Time-Frequency Methods and Phase Space.

[21] Papandreou G., Kokkinos I., Savalle P.A. Modeling local and global deformations in deep learning: Epitomic convolution, multiple instance learning, and sliding window detection. Proceedings of the IEEE Conference on Computer Vision and Pattern Recognition.

[22] Yu F., Koltun V. (2015). Multi-scale context aggregation by dilated convolutions. arXiv.

[23] Chen L.C., Papandreou G., Kokkinos I., Murphy K., Yuille A.L. (2014). Semantic image segmentation with deep convolutional nets and fully connected crfs. arXiv.

[24] Liu W., Anguelov D., Erhan D., Szegedy C., Reed S., Fu C.Y., Berg A.C. Ssd: Single shot multibox detector. Proceedings of the European Conference on Computer Vision.

[25] Dai J., He K., Li Y., Ren S., Sun J. Instance-sensitive fully convolutional networks. Proceedings of the European Conference on Computer Vision.

[26] Yang M., Yu K., Zhang C., Li Z., Yang K. DenseASPP for Semantic Segmentation in Street Scenes. Proceedings of the IEEE Conference on Computer Vision and Pattern Recognition.

[27] Vaswani A., Shazeer N., Parmar N., Uszkoreit J., Jones L., Gomez A.N., Kaiser L., Polosukhin I. (2017). Attention is all you need. Adv. Neural Inf. Process. Syst..

[28] Velickovic P., Cucurull G., Casanova A., Romero A., Lio P., Bengio Y. (2017). Graph attention networks. Stat.

[29] Wang X., Girshick R., Gupta A., He K. Non-local neural networks. Proceedings of the IEEE Conference on Computer Vision and Pattern Recognition.

[30] Huang Z., Wang X., Huang L., Huang C., Wei Y., Liu W. Ccnet: Criss-cross attention for semantic segmentation. Proceedings of the IEEE/CVF Conference on Computer Vision and Pattern Recognition.

[31] Hu J., Shen L., Sun G. Squeeze-and-excitation networks. Proceedings of the IEEE Conference on Computer Vision and Pattern Recognition.

[32] Cao Y., Xu J., Lin S., Wei F., Hu H. GCNet: Non-Local Networks Meet Squeeze-Excitation Networks and Beyond. Proceedings of the IEEE/CVF Conference on Computer Vision and Pattern Recognition.

[33] Ronneberger O., Fischer P., Brox T. U-net: Convolutional networks for biomedical image segmentation. Proceedings of the International Conference on Medical Image Computing and Computer-Assisted Intervention.

[34] He K., Zhang X., Ren S., Sun J. Deep residual learning for image recognition. Proceedings of the IEEE Conference on Computer Vision and Pattern Recognition.

[35] Wang Z., Bovik A.C., Sheikh H.R., Simoncelli E.P. (2004). Image quality assessment: From error visibility to structural similarity. IEEE Trans. Image Process..

[36] Mayer N., Ilg E., Hausser P., Fischer P., Cremers D., Dosovitskiy A., Brox T. A large dataset to train convolutional networks for disparity, optical flow, and scene flow estimation. Proceedings of the IEEE Conference on Computer Vision and Pattern Recognition.

[37] Deng J., Dong W., Socher R., Li L.J., Li K., Fei-Fei L. ImageNet: A large-scale hierarchical image database. Proceedings of the 2012 IEEE Conference on Computer Vision and Pattern Recognition.

[38] Geiger A., Lenz P., Urtasun R. Are we ready for autonomous driving?. the kitti vision benchmark suite. In Proceeding of the 2012 IEEE Conference on Computer Vision and Pattern Recognition.

[39] Geiger A., Lenz P., Stiller C., Urtasun R. (2021). Vision meets robotics: The kitti dataset. Int. J. Robot. Res..

[40] Li S., Wang X., Cao Y., Xue F., Yan Z., Zha H. Self-supervised deep visual odometry with online adaptation. Proceedings of the IEEE/CVF Conference on Computer Vision and Pattern Recognition.

[41] Zhan H., Garg R., Weerasekera C.S., Li K., Agarwal H., Reid I. Unsupervised learning of monocular depth estimation and visual odometry with deep feature reconstruction. Proceedings of the IEEE Conference on Computer Vision and Pattern Recognition.

[42] Li Y., Ushiku Y., Harada T. Pose graph optimization for unsupervised monocular visual odometry. Proceedings of the International Conference on Robotics and Automation.

[43] Li X., Hou Y., Wang P., Gao Z., Xu M., Li W. (2021). Transformer guided geometry model for flow-based unsupervised visual odometry. Neural Comput. Appl..

[44] Xue F., Wang Q., Wang X., Dong W., Wang J., Zha H. (2018). Guided feature selection for deep visual odometry. Proceeding of the Asian Conference on Computer Vision, Perth, Australia, 2–6 December 2018.

[45] Kuo X.Y., Liu C., Lin K.C., Lee C.Y. Dynamic Attention-based Visual Odometry. Proceedings of the 2020 IEEE/RSJ International Conference on Intelligent Robots and Systems.

[46] Gadipudi N., Elamvazuthi I., Lu C.-K., Paramasivam S., Su S. (2021). WPO-Net: Windowed Pose Optimization Network for Monocular Visual Odometry Estimation. Sensors.

[47] Eigen D., Puhrsch C., Fergus R. (2014). Depth map prediction from a single image using a multi-scale deep network. Adv. Neural Inf. Process. Syst..

[48] Ranjan A., Jampani V., Balles L., Kim K., Sun D., Wulff J., Black M.J. Competitive collaboration: Joint unsupervised learning of depth, camera motion, optical flow and motion segmentation. Proceedings of the IEEE/CVF Conference on Computer Vision and Pattern Recognition.

[49] Godard C., Mac Aodha O., Brostow G.J. Unsupervised monocular depth estimation with left-right consistency. Proceedings of the IEEE Conference on Computer Vision and Pattern Recognition.

[50] Pilzer A., Lathuiliere S., Sebe N., Ricci E. Refine and distill: Exploiting cycle-inconsistency and knowledge distillation for unsupervised monocular depth estimation. Proceedings of the IEEE/CVF Conference on Computer Vision and Pattern Recognition.

[51] Wong A., Soatto S. Bilateral cyclic constraint and adaptive regularization for unsupervised monocular depth prediction. Proceedings of the IEEE/CVF Conference on Computer Vision and Pattern Recognition.

